# Division of the Genio-hyo-glossi Muscles for Stammering

**Published:** 1841-11

**Authors:** Alex. J. Lizars

**Affiliations:** Lecturer on Anatomy and Opertive Surgery


*Division of the Genio-hyo-glossi Muscles for Stammering.* By Alex. J. Lizars, M. D., Lecturer on Anatomy and Opertive Surgery.—P. M., aged 35, had stammered from his infancy. The difficulty was evidently caused by spasmodic contraction of the muscles of the tongue and neck. The tongue, upon examination, was found to be shorter than natural. I operated in presence of my assistants, Messrs. Riccard and Hole, and two of my pupils, Messrs. Collyns and Tibbets.

The instruments employed were, a straight sharp-pointed bistoury; a curved probe-pointed bistoury, with the cutting edge about an inch long, the remainder of the blade being blunt; a four-headed sling, or roller, and a compress of lint.

The patient having been placed in the sitting posture, with the sharp-pointed bistoury I made a puncture, rather less than a quarter of an inch in length, through the integuments of the lower part of the chin, about an inch posterior to the symphysis. I then pushed the curved bistoury gently upwards and a little forwards, until I saw its probe elevating the mucous membrane of the floor of the mouth; placing the forefinger of my left hand upon the probe-point and mucous membrane, I turned the cutting edge of the instrument to the right, and divided the muscles of that side; the bistoury was then carefully brought back to the mesial line, and the other muscle having been divided in a similar manner, the instrument was withdrawn. The compress of lint was then placed on the wound, and the four-headed sling applied in the same way as is done for fracture of the lower jaw.

Very little blood was lost during the operation; and after its completion the hæmorrhage was entirely stopped by the compress and bandage. Every thing went on favorably; the bandage was removed on the third day, by which time the wound had healed; and the patient resumed his usual occupation on the fourth day.

Immediately after the operation the patient experienced no difficulty in speaking, and the same has continued since. Upon examining the mouth after removing the bandage, blood was observed beneath the mucous membrane in the line of the submaxillary ducts; this was absorbed by the tenth day, and the patient was completely cured.—*Lancet; Med. Exam.*

